# New insights on the supramolecular structure of highly porous core–shell drug nanocarriers using solid-state NMR spectroscopy†

**DOI:** 10.1039/c9ra07383c

**Published:** 2019-10-14

**Authors:** Marianna Porcino, Ioanna Christodoulou, Mai Dang Le Vuong, Ruxandra Gref, Charlotte Martineau-Corcos

**Affiliations:** CNRS, CEMHTI UPR 3079, Université d'Orléans 1d Avenue de la recherche scientifique 45071 Orléans France; ISMO, UMR 8214 CNRS, Université Paris Sud, Université Paris Saclay 91400 Orsay France ruxandra.gref@u-psud.fr; MIM, Institut Lavoisier de Versailles (ILV), UMR CNRS 8180, Université de Versailles St-Quentin en Yvelines (UVSQ) 45 Avenue des Etats-Unis 78035 Versailles Cedex France charlotte.martineau@uvsq.fr

## Abstract

Nano-sized metal–organic frameworks (nanoMOFs), with engineered surfaces to enhance the targeting of the drug delivery, have proven efficient as drug nanocarriers. To improve their performances a step further, it is essential to understand at the molecular level the interactions between the nanoMOF interfaces and both the surface covering groups and the drug loaded inside the micropores. Here we show how solid-state NMR spectroscopy allows us to address these issues in an aluminum-based nanoMOF coated and loaded with phosphorus-containing species.

## Abstract 2

Nano-sized metal–organic frameworks (nanoMOFs), with engineered surfaces to enhance the targeting of the drug delivery, have proven efficient as drug nanocarriers.

## Graphical abstract

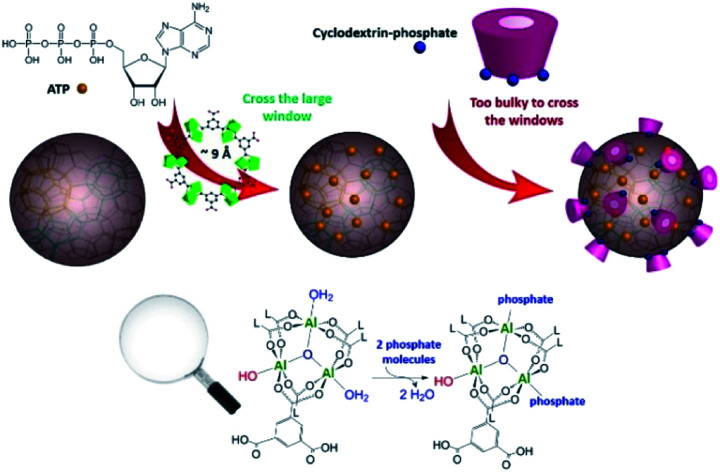

Since their recent discovery, iron-based nanoMOFs have attracted increasing interest due to their potential for medical applications, owing to the versatility of their structural features, *in vivo* biocompatibility and biodegradability, properties as contrast agents and possibility to tailor their surface functionalities.^1,2^ Among them, iron trimesate nanoMOF MIL-100(Fe) (MIL stands for Material of Institute Lavoisier) is among the most promising candidates for efficient drug incorporation by a one-step organic solvent-free method and for surface coating with cyclodextrin (CD) molecules which can be further engineered by linking phosphates (CD-P), targeting moieties or polymers.^3,4^ The interactions of surface-modified iron trimesate nanoMOFs with cancer cells were increased by means of these versatile “Lego”-type coatings.^3^ If the potential applications of the CD-P coated nanoMOFs have been demonstrated,^1,5^ little information is known about the interactions at the atomic level between the CD-P coating and the nanoparticle (NP) surface sites. It was hypothesized that, in contrast to drug molecules which cross the windows of the MOFs and adsorb inside the pores, CD-Ps are too bulky and supposed not to bypass windows of around 9 Å in diameter and effectively remain adsorbed onto the external surface (Fig. 1). Having a direct proof of this hypothesis could help guide further CD-P engineering. Understanding the interactions at the molecular level between the drug and the host solid is also crucial, as these interactions have a strong influence on the delivery processes. This study addresses these important aspects by using up-to-date complementary solid-state spectroscopic methodologies.

**Fig. 1:**
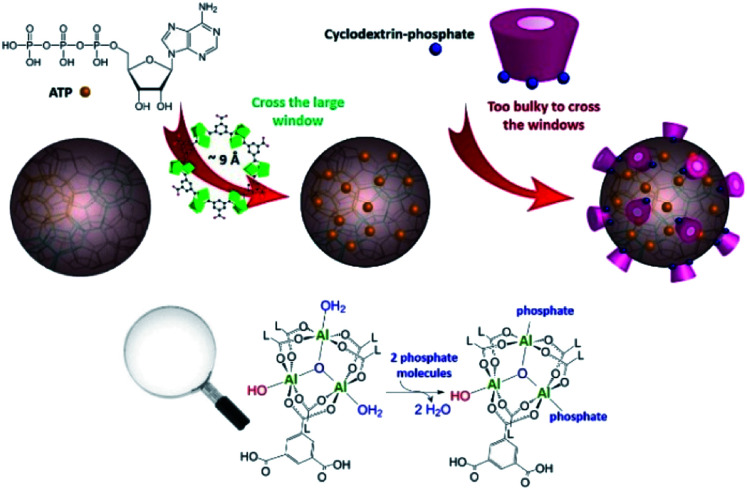
Upper panel: schematic representation of the highly porous MIL-100(Al) nanoparticles loaded with ATP and then coated with CD-P. Bottom panel: close up of Al trimers coordinated to trimesate ligands (L). Two molecules of bound water can be replaced either by phosphates from the drug or by phosphate grafted on the cyclodextrin molecules. Phosphates bound to CD can only access sites located close to the external MOF surface. CD dimensions: cage 6–6.5 Å, external diameter 15.4 Å, height ∼8 Å. ATP molecule has about 7 Å radius.

In the past decades, magic-angle spinning (MAS) solid-state nuclear magnetic resonance (ssNMR) spectroscopy, sensitive to short-range order, has proven an essential technique to get information about the structure of MOFs,^6^ including drug-carrier MOFs.^7^ Although it appeared ideally suited to address the questions mentioned above, we faced the presence of strong paramagnetic centers (Fe^3+^ cations) that severely reduces the relaxation times hence the amount of available information on the NMR spectra. We therefore have chosen to focus our study on the diamagnetic analogue of nanoMIL-100(Fe), namely nanoMIL-100(Al). The MIL-100(Al) topology^8^ is similar to that of MIL-100(Fe),^9^ it was shown that large amounts of drugs can be incorporated in this material, and that the NP surface (Fig. 1) can also be efficiently coated by CD-P.^3^ Drug of interest here is adenosine triphosphate (ATP), a neurotransmitter with a crucial role in metabolism, which is believed to have a strong affinity with the aluminium species of the nanoMOF framework.

During our investigation, we were confronted to several challenges, including: (i) the complexity of the system, which yields broad overlapping ^1^H MAS NMR spectra even at high field (20 T) and fast-MAS (60 kHz, Fig. S1, ESI†), and (ii) the low quantity of the surface species despite the reduced size (150 nm) of the particles. To circumvent the latter difficulty, surface enhanced solid-state NMR spectroscopy such as dynamic nuclear polarization (DNP) methods have been developed. They have proven their efficiency to detect the species present at the external surfaces of silica nanoparticles^10^ and the applicability of the method for MOFs was shown.^11^ However, DNP-MAS requires the use of a heterogeneous radical, solvent, low temperature (100 K), hence it does not represent the state of NPs as they can potentially be administered *in vivo* in patients. Therefore, we chose to work at room temperature and with simpler MAS NMR methods, namely ^27^Al, ^31^P and 2D ^27^Al–^31^P correlation spectroscopy, that did not involve the use of external molecules such as solvents or radicals.

The ^27^Al MAS NMR spectra of pristine and CD-P surface coated nanoMIL-100(Al) (Fig. S2, ESI†) are essentially similar despite the use of high magnetic field (20 T). For quadrupolar nuclei such as ^27^Al, greater resolution can often be obtained using the multiple-quantum MAS (MQ-MAS) NMR experiment that separates the aluminium species in the indirect (vertical) dimension. The ^27^Al MQMAS NMR spectrum of the nanoMIL-100(Al) recorded at moderate static magnetic field (9.4 T, Fig. S2, ESI†) is similar to that reported earlier for micron-sized MIL-100(Al) recorded at 11.7 T.^12^ The ^27^Al MQMAS NMR spectrum of nanoMIL-100(Al) recorded at 20 T interestingly has increased resolution which reveals the presence of an additional peak located at around −5 ppm (Fig. 2a). While the bulk Al signals remain similar, the new aluminium sites are in turn slightly modified when the NPs are coated with the CD-P (Fig. 2b), which indicates (i) that this aluminium resonance is due to the Al atoms present at or slightly below the surface of the NPs since CD-P is too bulky (see Fig. 1) to penetrate the open 3D-MOF structure, (ii) the shift of the resonance upon coating results from interactions between some of the surface aluminium species of the NPs and the CD-P coating. In the drug loaded samples (Fig. 3c and d), the bulk Al signals are more affected, indicating the successful incorporation of the drug inside the micropores of the nanoMOF.

**Fig. 2:**
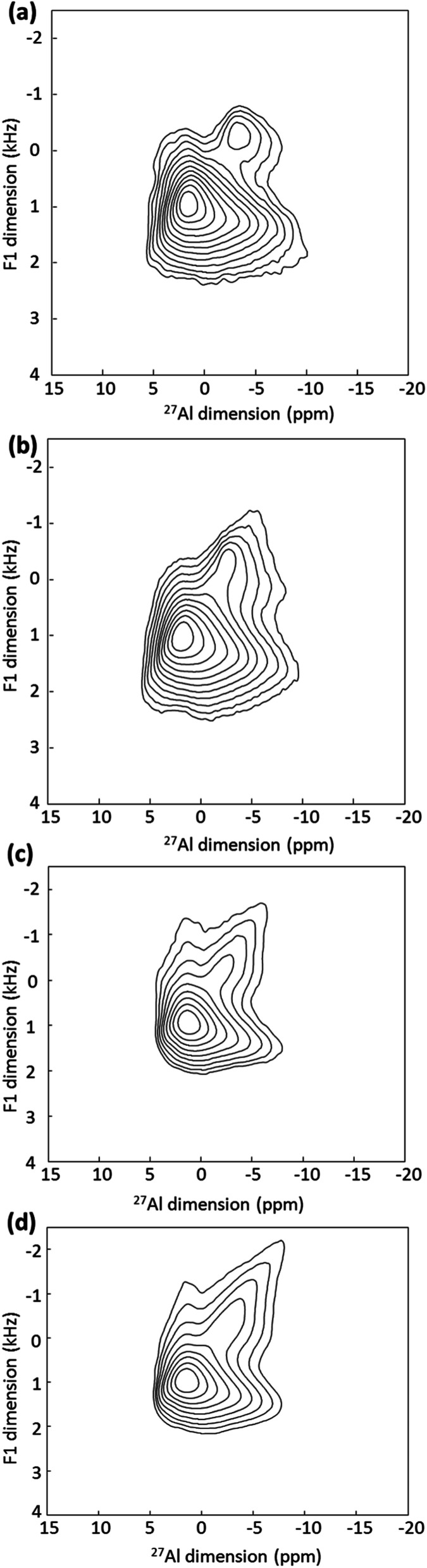
^27^Al MQMAS NMR spectra of (a) pristine nanoMIL-100(Al) and nanoMIL-100(Al) (b) coated with CD-P, (c) loaded with ATP and (d) loaded with ATP and coated with CD-P.

**Fig. 3:**
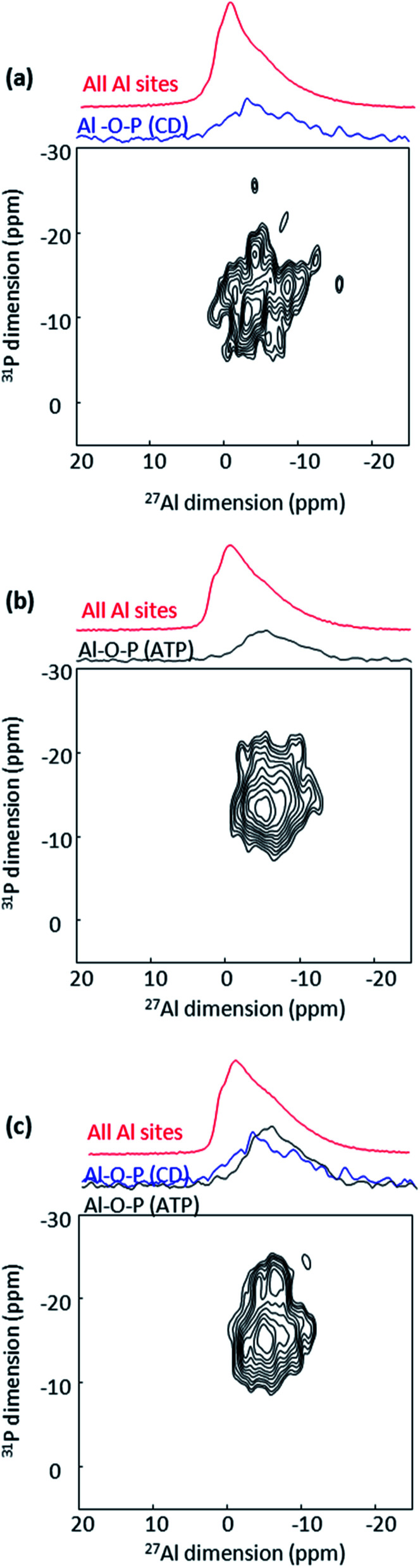
^27^Al{^31^P} MAS D-HMQC NMR spectra of CD-P coated (a), ATP loaded (b) and CD-P coated ATP loaded nanoMIL100(Al) (c). The top blue spectra are the full projections on the horizontal dimension for the surface sites, the black spectra are the full projection for the interphase sites, while the red spectra are the MAS NMR spectra shown for comparison.

To understand further these interactions, through-space ^1^H–^27^Al two-dimensional (2D) MAS NMR experiments were performed in an attempt to correlate the protons of the CD-P that are in close proximity to the aluminium species. However the ^1^H NMR spectrum is dominated by the signal of proton of the linkers, hence only the ^1^H–^27^Al correlations from the bulk of the NPs were detected (Fig. S3, ESI†).

The ^31^P MAS NMR signatures of ATP and CD-P are well distinct (Fig. S5, ESI†). ^31^P–^31^P double-quantum single-quantum (DQ-SQ) NMR experiments were performed, which allow probing the spatial proximity between the phosphate groups. In CD-P, the ^31^P DQ-SQ NMR spectrum shows the phosphate groups grafted on the CD cavity. In ATP, such a spectrum allows the distinction of the middle phosphate of the tri-phosphate chain, which is the only ^31^P resonance that has two other P neighbours (Fig. S4, ESI†). In both ATP-loaded and ATP-loaded and coated nanoMIL-100(Al), the ^31^P DQ-SQ NMR correlations are similar to pure ATP, showing that the triphosphate moieties are preserved after loading (Fig. S6, ESI†). One can notice a shift of the ^31^P resonances, which very likely indicates strong interaction with the framework aluminium sites.

To go further in the characterization, we took advantage of the heteronuclei present in the CD-P coated nanoMIL-100(Al): ^27^Al, arising solely from the nanoMOF and ^31^P, arising solely from the CD-P coating. By recording 2D NMR spectrum between ^31^P and ^27^Al nuclei, the surface aluminium species of the NPs in close proximity to the CD-P and the bulk aluminium sites located in the vicinity of the loaded drug are expected to be selectively detected. Because of the short ^27^Al transverse relaxation time *T*_2_ (Table S1, ESI†), we could not use the through-bond heteronuclear multiple-quantum (J-HMQC) experiment that requires recoupling time in the 8 ms range for optimum efficiency.^13^ Instead we employed the through-space dipolar-based version (D-HMQC), for which the optimum dipolar recoupling requires shorter recoupling time.^14^ The recoupling time was kept short enough to ensure that only the Al in very close proximity to the ^31^P were selected. The resulting 2D ^27^Al–^31^P MAS NMR spectrum of CD-P-coated nanoMIL-100(Al) is shown Fig. 3a. The ^27^Al NMR signal is at −5 ppm (*i.e.*, chemical shift similar to that of the surface peak observed in the MQMAS NMR spectra Fig. 2), which indicates that the observed surface Al species in the vicinity of the CD-P are in six-fold coordination. The ^27^Al chemical shift is lower than the ^27^Al chemical shifts in pristine MIL-100(Al) which range between 1.1 and 3.4 ppm.^12^ It is close to chemical shift observed in aluminophosphate, hence we can hypothesize that the six-fold coordination is preserved by the formation of an Al–O–P bond between the Al surface sites of the nanoMOF and the terminal phosphate groups of the CD-P, which very likely substitute a water molecule (Fig. 1).

A similar six-fold coordinated ^27^Al resonance around −7 ppm is observed in the ATP loaded nanoMIL-100(Al) 2D ^27^Al–^31^P D-HMQC MAS NMR spectrum (Fig. 3b), which suggests a close proximity (formation of a Al–O–P chemical bond) between Al species of the MOF framework and the terminal phosphate of the drug, which ^31^P resonance is located at −10 ppm. This ^27^Al resonance is the signature of the grafted aluminium sites inside the pores of the MOF. Correlation of lower intensity is also observed between these Al species and the middle P of the triphosphate chain (−20 ppm), in agreement with longer Al–P distance. Here again, the ATP very likely replaces a water molecule from the Al tricluster.

Finally, we performed the same 2D ^27^Al–^31^P NMR experiment in the target CD-P coated nanoMIL-100(Al) loaded with ATP (Fig. 3c). The ATP content is much higher than the CD-P loading, hence the spectrum is dominated by the Al–O-ATP interaction. However, the ^31^P chemical shift expands to the higher ppm region (−5 to −10 ppm), characteristic of the CD-P coating. The similarity of the spectra in Fig. 3b and c clearly confirms that the CD-P coating on the external surface of the ATP-loaded nanoMOF did not affect the Al–O-ATP bond formed inside the MOF cavities.

In conclusion, by taking advantage of distinct heteroatoms present in an aluminium-based nanoMOF (^27^Al) which surface was coated by bulky phosphated CD groups (^31^P), we could circumvent the low resolution of ^1^H MAS NMR spectroscopy and provide for the first time a ^27^Al NMR signature at room temperature of aluminium species present at the surface or below the surface of MOF NPs and their interaction (*i.e.*, the formation of Al–O–P covalent bonds) with the CD-P coating. The formation of this strong covalent bonding between the CD and the MOF might be the key to ensure high stability of the core–shell NP *in vivo*. Using the same NMR methodology, we have shown the strong interaction of the triphosphate-drug ATP with the Al atoms of the MOF framework. Finally, we have shown the successful CD-P coating in a ATP-loaded nanoMIL-100(Al), which did not modify the MOF-drug interactions. This set of ssNMR experiment represents an essential characterization tool to guide towards more efficient surface modifications of these NPs, better targeting drugs (mono, di or tri-phosphate) and to help in understanding the effect of drug incorporation on the surface state of the nanoMOF. It could also be very useful in the investigation of nanoMIL-100(Al) NPs degradation in physiological medium (phosphate buffer) in which Al–O–P interactions might occur and be at the origin of the drug release processes. The methodology presented here consisting in covering the surface of porous NPs by bulky groups (unable to penetrate in the porosity of the particles) containing heteroatoms easily accessible by ssNMR (like ^31^P or ^19^F) could also become a general strategy to probe surface species of MOF NPs without need of expensive DNP-MAS experiments.

## Conflicts of interest

There are no conflicts to declare.

## Supplementary Material

RA-009-C9RA07383C-s001

## References

[cit1] Horcajada P., Chalati T., Serre C., Gillet B., Sebrie C., Baati T., Eubank J. F., Heurtaux D., Clayette P., Kreuz C., Chang J. S., Hwang Y. K., Marsaud V., Bories P. N., Cynober L., Gil S., Ferey G., Couvreur P., Gref R. (2010). Nat. Mater..

[cit2] Baati T., Njim L., Neffati F., Kerkeni A., Bouttemi M., Gref R., Najjar M. F., Zakhama A., Couvreur P., Serre C., Horcajada P. (2013). Chem. Sci..

[cit3] Agostoni V., Horcajada P., Noiray M., Malanga M., Aykac A., Jicsinszky L., Vargas-Berenguel A., Semiramoth N., Daoud-Mahammed S., Nicolas V., Martineau C., Taulelle F., Vigneron J., Etcheberry A., Serre C., Gref R. (2015). Sci. Rep..

[cit4] Aykac A., Noiray M., Malanga M., Agostoni V., Casas-Solvas J. M., Fenyvesi E., Gref R., Vargas-Berenguel A. (2017). Biochim. Biophys. Acta.

[cit5] Simon-Yarza T., Baati T., Paci A., Lesueur L. L., Seck A., Chiper M., Gref R., Serre C., Couvreur P., Horcajada P. (2016). J. Mater. Chem. B.

[cit6] Martineau-Corcos C. (2018). Curr. Opin. Colloid Interface Sci..

[cit7] Devautour-Vinot S., Martineau C., Diaby S., Ben-Yahia M., Miller S., Serre C., Horcajada P., Cunha D., Taulelle F., Maurin G. (2013). J. Phys. Chem. C.

[cit8] Ferey G., Serre C., Mellot-Draznieks C., Millange F., Surble S., Dutour J., Margiolaki I. (2004). Angew. Chem., Int. Ed..

[cit9] Volkringer C., Popov D., Loiseau T., Ferey G., Burghammer M., Riekel C., Haouas M., Taulelle F. (2009). Chem. Mater..

[cit10] Rossini A. J., Zagdoun A., Lelli M., Lesage A., Copéret C., Emsley L. (2013). Acc. Chem. Res..

[cit11] Rossini A. J., Zagdoun A., Lelli M., Canivet J., Aguado S., Ouari O., Tordo P., Rosay M., Maas W. E., Coperet C., Farrusseng D., Emsley L., Lesage A. (2012). Angew. Chem., Int. Ed..

[cit12] Haouas M., Volkringer C., Loiseau T., Ferey G., Taulelle F. (2011). J. Phys. Chem. C.

[cit13] Massiot D., Fayon F., Alonso B., Trebosc J., Amoureux J. P. (2003). J. Magn. Reson..

[cit14] Trebosc J., Hu B., Amoureux J. P., Gan Z. (2007). J. Magn. Reson..

