# Supplementary material for: New insights on the supramolecular structure of highly porous core–shell drug nanocarriers using solid-state NMR spectroscopy
Source: RSC Adv. 2019 Oct 14;9(56):32472–5. doi: 10.1039/c9ra07383c (PMC9072847; doi:10.1039/c9ra07383c)
Supplement: RA-009-C9RA07383C-s001 [file RA-009-C9RA07383C-s001.pdf]

Electronic Supplementary Material (ESI) for RSC Advances.
This journal is © The Royal Society of Chemistry 2019

New insights on the supramolecular structure of highly porous core-shell drug nanocarriers using solid-state NMR spectroscopy.

Marianna Porcino, Ioanna Christodoulou, Mai Vuong Dang Le, Ruxandra Gref and Charlotte Martineau-Corcos

Electronic Supplementary Information

Experimental Section

Fig. S1. TEM images of nanoMIL-100(Al).

Fig. S2. TEM images of CD-P-coated nanoMIL-100(Al).

Fig. S3. TEM images of nanoMIL-100(Al) degraded 48 hours in PBS solution.

Fig. S4. ^{1}H 1D and ^{1}H-^{1}H 2D MAS NMR spectra

Fig. S5. ^{27}Al MAS and MQMAS NMR spectra

Table S1. ^{27}Al T_2 values

Fig. S6. ^{27}Al{^{1}H} MAS *D*-HMQC NMR spectra

Fig. S7. ^{31}P-^{31}P MAS DQ-SQ NMR spectrum of pure ATP

Fig. S8. ^{31}P CPMAS NMR spectra

Fig. S9. ^{31}P-^{31}P MAS DQ-SQ NMR spectra of pure loaded or coated nanoMIL-100(Al)

Experimental Section

Synthesis

Al MIL(100) NPs were prepared and characterized as previously described.[1] The crystalline particles had a mean hydrodynamic diameter of 160 ± 15 nm, BET surface area of 1670±20 m^2/g. ATP was loaded according to previous organic solvent-free method reported in the case of other phosphorylated drugs.[2-4] Loadings reached 20 wt%.

Elemental analysis

Elemental analysis was performed to quantify the C and H elemental weight contents in the MOF particles loaded or not with ATP after sample combustion at 1050°C using an Elemental Analyzer (Perkin-Elmer 2400 CHNS/O Series II System).

Transmission Electron Microscopy (TEM)

Morphology and size of the solids were observed by Transmission Electron Microscopy (TEM), under a JEOL JEM-1400 microscope with acceleration voltage of 120 kV. Nanoparticle suspensions were deposited on copper grids, left to dry and observed without further staining.

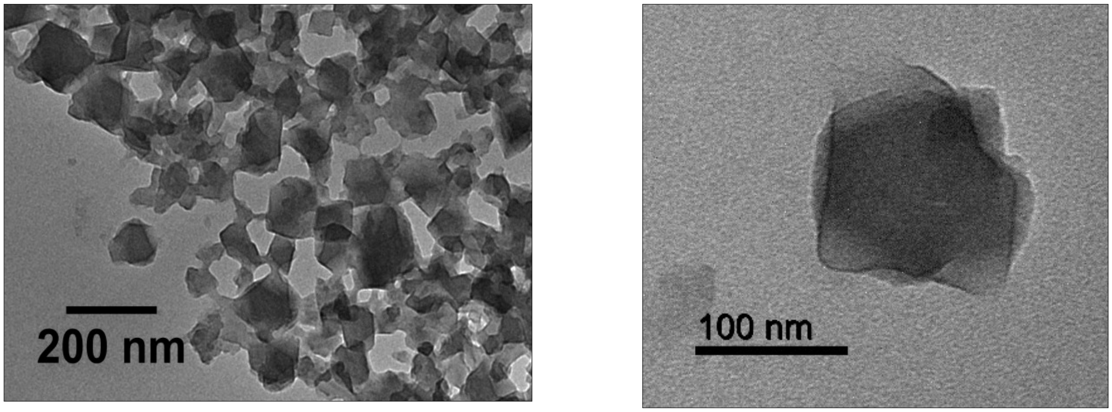

Figure S1. TEM images of nanoMIL-100(Al).

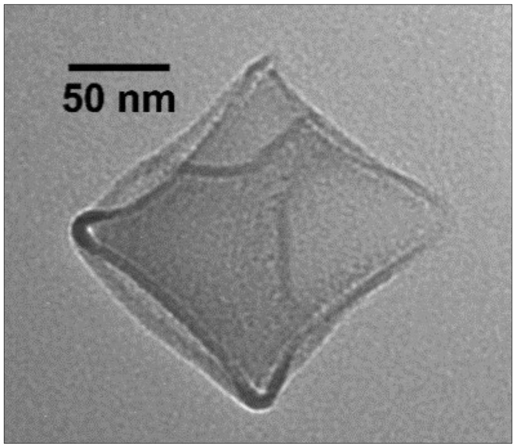

Figure S2. TEM images of CD-P coated nanoMIL-100(Al).

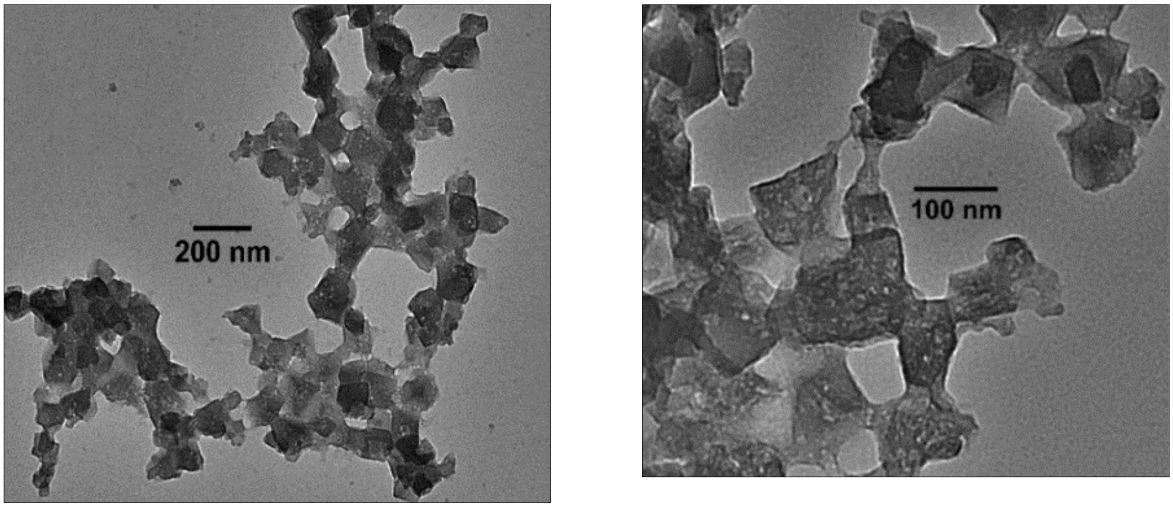

Figure S3. TEM images of nanoMIL-100(Al) degraded 48 hours in PBS solution.

All TEM imaged show no dissolution of the nanoparticles. Degradation in PBS occurs through the formation of holes in the NPs.

Solid-state NMR Spectroscopy

The ^{27}Al MAS and MQMAS NMR spectra were recorded at 20 T using a H/X/Y 3.2 mm double-resonance probehead, spinning at 20 kHz MAS frequency. The z-filter MQMAS pulse sequence was used, with radiofrequency (RF) field for the excitation and reconversion of the triple-quantum coherence. The ^{27}Al{^{31}P} *D*-HMQC NMR spectrum was recorded at 17.6 T using a 4 mm ^{1}H-^{31}P-^{27}Al triple resonance probe spinning at 14 kHz MAS frequency. The symmetry-based R412 scheme[5] was used to recouple the Al-P dipolar interaction. The rotor-synchronized recoupling time was set to 1.7 ms. ^{27}Al double-frequency sweep (DFS) was applied to boost the ^{27}Al

magnetization. Recycling delay was set to 0.25 s. 20 t_1 slices were recorded with 32432 (for the coated material) and 28800 (for the ATP-loaded ones) transients each, leading to a total of experimental time of about 45 hours. All spectra are treated with 200 Hz LB apodization in the F2 dimension. The ^{27}Al and ^{31}P chemical shifts are referenced to $Al(NO_3)_3$ and H_3PO_4 solution at 0 ppm, respectively. All spectra were analysed using the Dmfit software.[6]

The ^{1}H MAS NMR spectra were recorded at a magnetic field of 20 T, using a Bruker 850 MHz WB NMR spectrometer and a HXY 1.3 mm probe in double mode. The spectra were acquired using a Hahn echo pulse sequence, with a 90° pulse duration of 3.5 μs, an inter-pulse delay synchronized with one rotor period and a spinning rate of 60 kHz. The recycle delay was set to 5 s and 16 transients were recorded for each sample. The ^{1}H chemical shifts were referenced to TMS at 0 ppm.

The ^{27}Al MAS NMR spectra were recorded at the same magnetic field of the ^{1}H spectra, using a HXY 3.2 mm probe in double mode, with a spinning rate of 20 kHz. The recycle delay was set to 0.3 s, the 90° pulse to 2.2 μs with a RF field of 38 kHz. The ^{27}Al chemical shifts are referenced to $Al(NO_3)_3$ solution at 0 ppm.

The ^{27}Al{^{1}H} *D*-HMQC (dipolar-Heteronuclear multiquantum correlation) 2D experiment is performed under a MAS frequency of 60 kHz in a 1.3 mm probe using the same spectrometer mentioned before. $R4^2_1$ was used as the recoupling sequence in order to reintroduce ^{1}H-^{27}Al heteronuclear dipolar interactions, with 1.4 ms of recoupling time. 80 t_1 slices with 1024 transients were co-added. The States-TPPI procedure provides a phase sensitive 2D NMR spectrum.

The ^{27}Al MQMAS (Multi-Quantum Magic-Angle Spinning) was carried out at 9.4 T (Larmor Frequency of 104 MHz), using a HXY 2.5 mm probe in double mode with a spinning rate of 25 kHz, using a triple-quantum z-filtered pulse sequence and is shown after a shearing transformation.

The ^{31}P cross-polarization experiments were recorded on a 9.4 T magnet (^{1}H and ^{31}P Larmor frequency of 400 and 160 MHz, respectively) with a Bruker spectrometer, using a 4 mm double resonance probe and spinning at 14 kHz MAS frequency. The ^{31}P chemical shifts are referenced to H_3PO_4 solution at 0 ppm. Recycling delay was set to 5 s, the initial 90° pulse on ^{1}H to 4 μs with a RF field of 62 kHz and the contact time was set to 5 ms. ^{1}H SPINAL-64 decoupling was applied during the ^{31}P acquisition.

The ^{31}P-^{31}P DQ-SQ MAS spectra were recorded on the same 9.4 T spectrometer, using the same probe and the same spinning speed as mentioned before. The INADEQUATE pulse sequence was used with a recoupling delay of 7 ms, for a total experimental time of 4 hours. ^{1}H SPINAL-64 decoupling was applied

during recoupling time and acquisition with RF fields of 70 kHz and 79 kHz respectively. The S_3 pulse sequence was used with a recoupling delay of 2.8 ms. ^{1}H CW (RF field of 52 kHz) and SPINAL-64 (RF field of 70 kHz) decoupling were applied during recoupling time and acquisition respectively. Using a recycling delay of 4 s, 60 t_1 slices with 192-960 transients were co-added, leading to a total of experimental times of 16-64 hours. For both INADEQUATE and S_3, the sensitivity was boosted by an initial $^1H \rightarrow ^{31}P$ CP block.

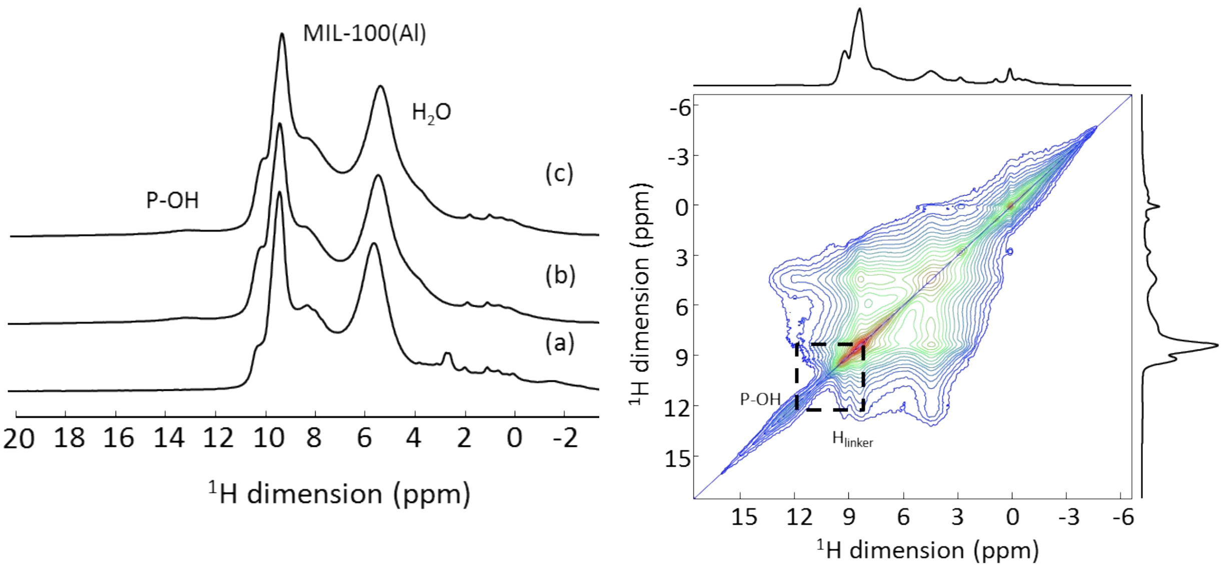

Figure S4. Left: ^{1}H MAS NMR spectra of (a) nanoMIL-100(Al), (b) ATP-loaded nanoMIL-100(Al) and (c) CD-P coated ATP-loaded nanoMIL-100(Al). Right: 2D ^{1}H-^{1}H spin diffusion NMR spectrum of CD-P coated ATP-loaded nanoMIL-100(Al). The dash lines indicate the spatial proximity between a phosphate proton and a proton from the linker of the MOF.

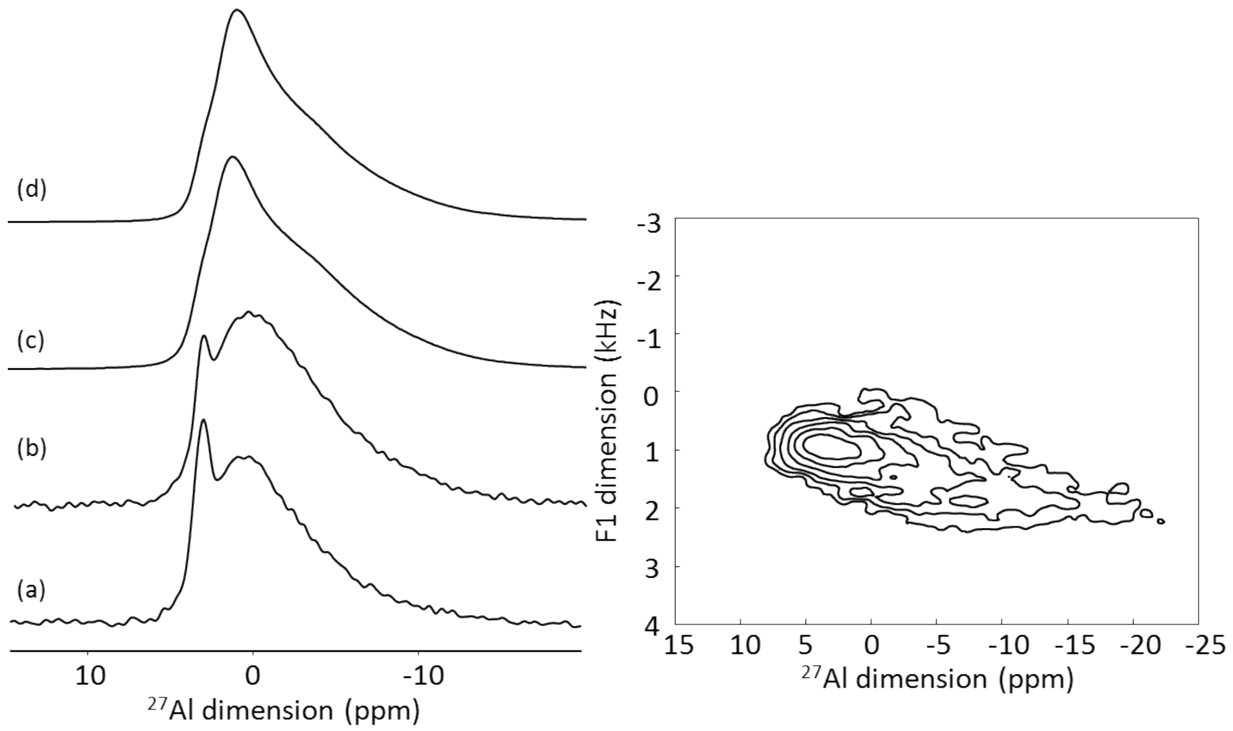

Figure S5. Left: ^{27}Al MAS NMR spectra of (a) nanoMIL-100(Al), (b) CD-P coated nanoMIL-100(Al), (c) ATP-loaded nanoMIL-100(Al) and (d) CD-P coated ATP-loaded nanoMIL-100(Al) recorded at 20.0 T. Right: ^{27}Al MQMAS NMR spectrum of nanoMIL-100(Al) recorded at 9.4 T.

The ^{27}Al MQMAS NMR spectrum of nanosized MIL-100(Al) is similar to the one previously reported in the literature for micro sized MIL-100(Al)[7] and display resonances in the chemical shift range of the six-coordinated aluminum atom (0 to – 10 ppm).

Sample	^{27}Al T_2 (ms, ±0.1)
MIL100(Al)	3.8
MIL100(Al)@ATP	3.4
CD-P coated MIL100(Al)	3.8
CD-P coated MIL100(Al)@ATP	3.5

Table S1. ^{27}Al T_2 measured from a spin echo experiment of the samples studied.

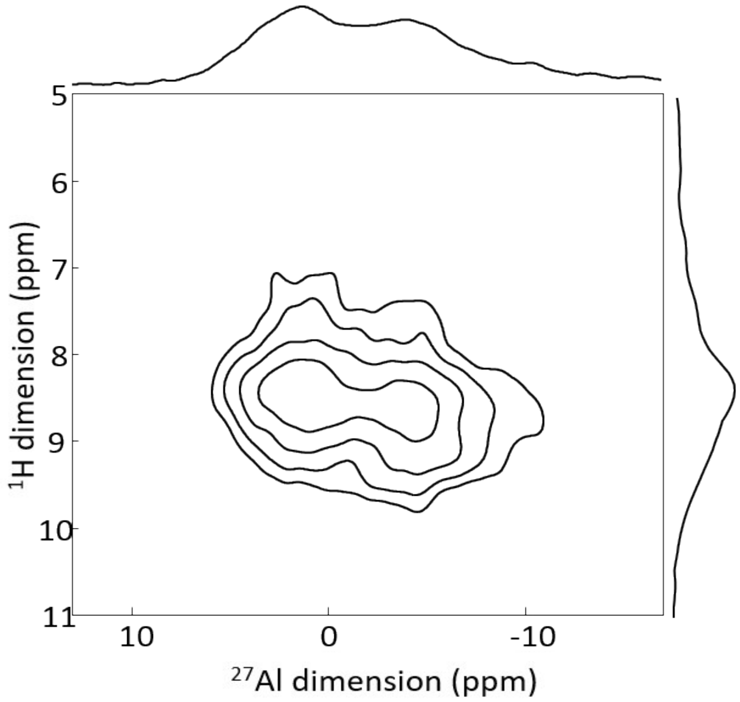

Figure S6. ^{27}Al{^{1}H} MAS *D*-HMQC NMR spectrum of CD-P coated MIL-100(Al). Only a correlation between the ^{27}Al signal and the protons of the linkers is observed.

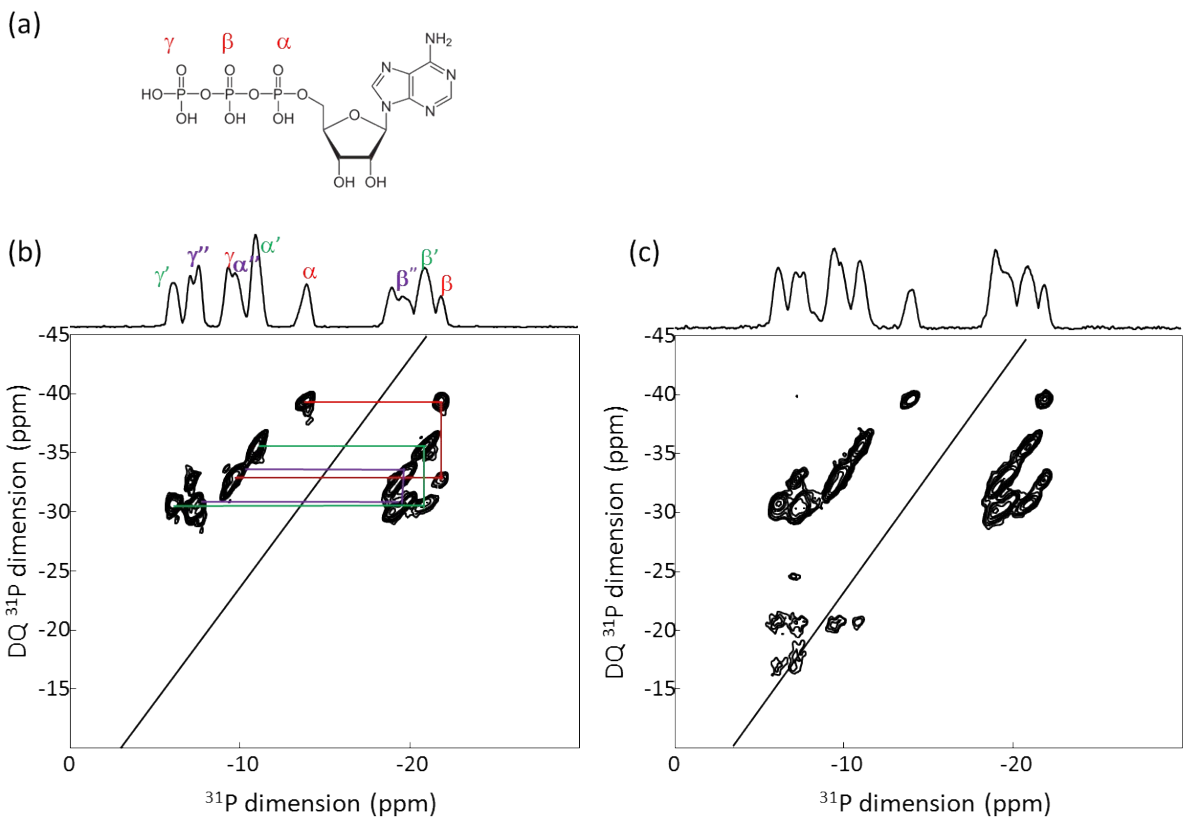

Figure S7. ^{31}P-^{31}P MAS DQ-SQ NMR spectrum of ATP recorded at 9.7 T with CP-INADEQUATE (left) and S_3 (right) pulse sequence. The lines represent the different hydrated forms of ATP present in the commercial product. Additional peaks are seen on the S_3 NMR spectrum (dipolar-based), which are due to longer range interactions.

In the 2D ^{31}P-^{31}P homonuclear correlation experiments (*J*-type and through space-proximity), the β-phosphorus atom (the middle of the tri-phosphate) is identified as it is the only one having correlation with two other P atoms. The commercial ATP is a mixture of several phases that differ by their hydration states. Additionally, the through-space correlation experiment (right part of Figure S4) gives the opportunity to observe the inter-molecular interactions.

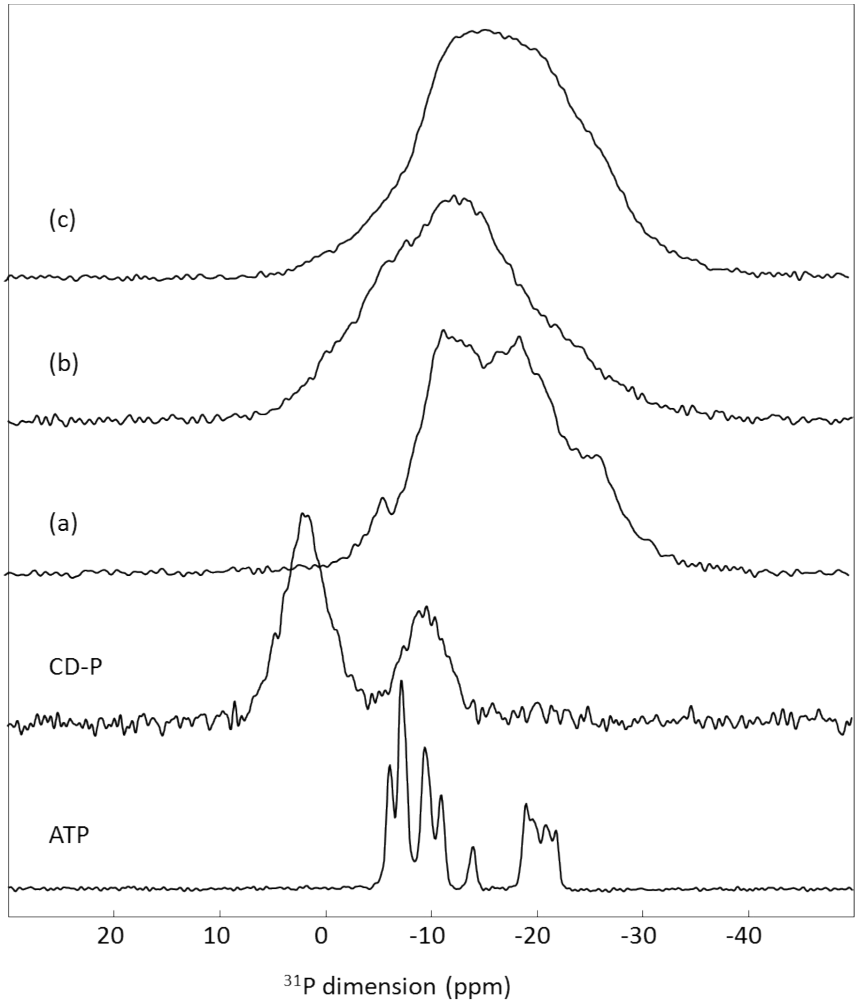

Figure S8. ^{31}P CPMAS NMR spectra of pure ATP, CD-P, (a) ATP loaded nanoMIL-100(Al), (b) CD-P coated nanoMIL-100 and (c) CD-P coated ATP loaded nanoMIL-100.

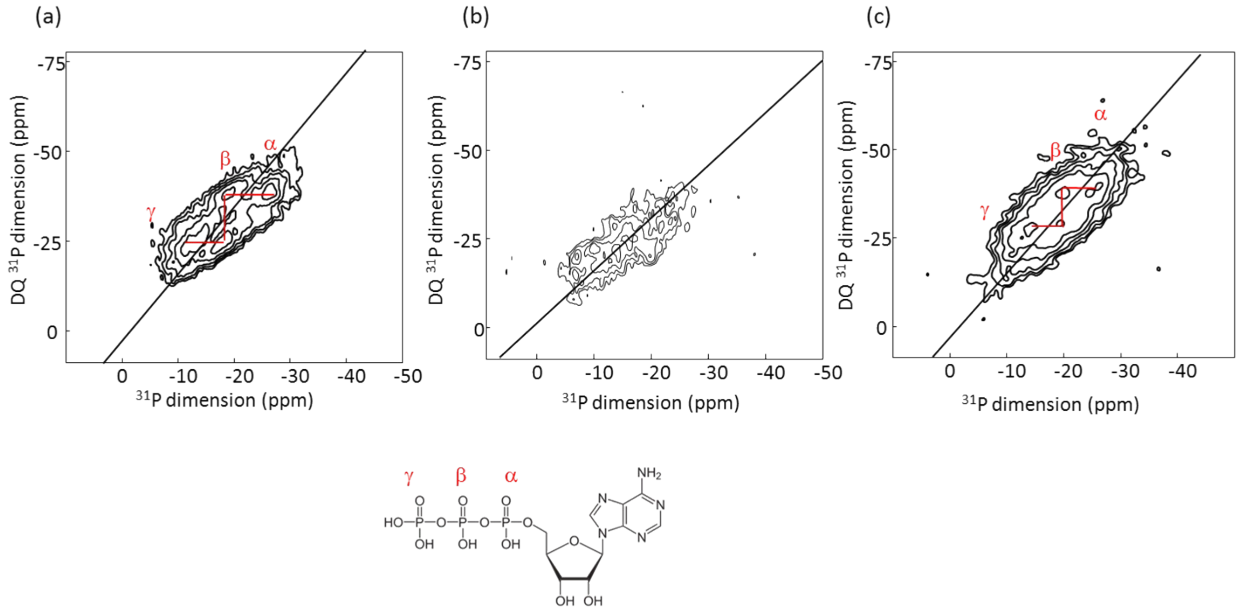

Figure S9. ^{31}P-^{31}P DQ-SQ MAS NMR spectra of (a) ATP loaded nanoMIL-100(Al), (b) CD-P coated nanoMIL-100 and (c) CD-P coated ATP loaded nanoMIL-100. Below is shown the ATP molecule with the triphosphate groups labeled.

Similar to the experiment performed on the pure ATP, the middle β-phosphate is identified as it is linked to two other phosphorus atoms. The triphosphate structure is preserved once the ATP is loaded in the pores of the nanoMOF. The ^{31}P resonance at *ca* – 15 ppm shows correlation with the aluminum atoms in the ^{27}Al-^{31}P *D*-HMQC NMR spectrum (see main text). It therefore corresponds to the terminal phosphate group.

References

1. V. Agostoni, P. Horcajada, M. Noiray, M. Malanga, A. Aykac, L. Jicsinszky, A. Vargas-Berenguel, N. Semiramoth, S. Daoud-Mahammed, V. Nicolas, C. Martineau, F. Taulelle, J. Vigneron, A. Etcheberry, C. Serre, R. Gref, *Sci. Reports* 2015, **5**, 7925.

2. V. Rodriguez-Ruiz, A. Maksimenko, R. Anand, S. Monti, V. Agostoni, P. Couvreur, M. Lampropoulou, K. Yannakopoulou, R. Gref, *J. Drug Target.*, 2015, **23**, 759.

3. V. Agostoni, R. Anand, S. Monti, S. Hall, G. Maurin, P. Horcajada, C. Serre, K. Bouchemal, R. Gref, *J. Mater. Chem. B*, 2013, **1**, 4231.

4. V. Agostoni, T. Chalati, P. Horcajada, H. Willaime, R. Anand, N. Semiramoth, T. Baati, S. Hall, G. Maurin, H. Chacun, K. Bouchemal, C. Martineau, F. Taulelle, P. Couvreur, C. Rogez-Kreuz, P. Clayette, S. Monti, C. Serre, R. Gref, *Adv. Healthcare Mater.*, 2013, **2**, 1630.
5. A. Brinkmann, A.P.M. Kentgens *J. Am. Chem. Soc.*, 2006, **128**, 14758.
6. D. Massiot, F. Fayon, M. Capron, I. King, S. Le Calvé, B. Alonso, J. O. Durand, B. Bujoli, Z. Gan, G. Hoatson, *Magn. Reson. Chem.*, 2002, **40**, 70.
7. M. Haouas, C. Volkringer, T. Loiseau, G. Ferey, F. Taulelle, *J. Phys. Chem. C*, 2011, **115**, 17934.
